# Cigarette smoke affects dendritic cell maturation in the small airways of patients with chronic obstructive pulmonary disease

**DOI:** 10.3892/mmr.2014.2759

**Published:** 2014-10-23

**Authors:** SHI-XIA LIAO, TING DING, XI-MIN RAO, DE-SHENG SUN, PENG-PENG SUN, YA-JUN WANG, DAN-DAN FU, XIAO-LI LIU, YAO OU-YANG

**Affiliations:** 1 Department of Respiratory Medicine, Affiliated Hospital of Zunyi Medical College, Zunyi, Guizhou 563003, P.R. China; 2 Department of Osteopathy, Affiliated Hospital of Zunyi Medical College, Zunyi, Guizhou 563003, P.R. China; 3 Department of Oncology, Affiliated Hospital of Zunyi Medical College, Zunyi, Guizhou 563003, P.R. China

**Keywords:** chronic obstructive pulmonary disease, reverse transcription polymerase chain reaction, chemokine receptor type 7, dendritic cells, cluster of differentiation 83, cluster of differentiation 1a

## Abstract

The aim of the present study was to characterize and quantify the numbers and expression levels of cells markers associated with dendritic cell (DC) maturation in small airways in current smokers and non-smokers with or without chronic obstructive pulmonary disease (COPD). Lung tissues from the following 32 patients were obtained during resection for lung cancer: Eight smokers with COPD, eight non-smokers with COPD, eight current smokers without COPD and eight non-smokers without COPD, serving as a control. The tissue sections were immunostained for cluster of differentiation (CD)83^+^ and CD1a^+^ to delineate mature and immature DCs, and chemokine receptor type 7 (CCR7^+^) to detect DC migratory ability. Myeloid DCs were collected from the lung tissues, and subsequently the CD83^+^ and CCR7^+^ expression levels in the lung myeloid DCs were detected using flow cytometry. The expression levels of CD83^+^, CD1a^+^ and CCR7^+^ mRNA in total lung RNA were evaluated by reverse transcription quantitative polymerase chain reaction (RT-qPCR). Evident chronic bronchitis and emphysema pathological changes were observed in the lung tissues of patients with COPD. The results revealed that the numbers of CD83^+^ and CCR7^+^ DCs were reduced but the numbers of CD1a^+^ DCs were significantly increased in the COPD group as compared with the control group (P<0.05, respectively). Using RT-qPCR, the expression levels of CCR7^+^ and CD83^+^ mRNA were found to be reduced in the smokers with COPD as compared with the non-smokers without COPD group (P<0.05, respectively). Excessive local adaptive immune responses are key elements in the pathogenesis of COPD. Cigarette smoke may stimulate immune responses by impairing the homing of airway DCs to the lymph nodes and reduce the migratory potential of DCs. The present study revealed that COPD is associated with reduced numbers of mature CD83^+^ DCs and lower CCR7^+^ expression levels in small airways.

## Introduction

Chronic obstructive pulmonary disease (COPD) is a common and frequently occurring disease of the respiratory system, with high morbidity and mortality rates (1). The exact cause of COPD is not currently clear, although COPD is widely considered to be associated with an abnormal inflammatory response of the lungs to noxious gases or particles, mainly due to cigarette smoke (CS) (2). In the current definition of COPD, there appears to be an immune basis for the abnormal pattern of inflammation (3). An increasing number of studies have indicated that in COPD, the numbers of macrophages, T lymphocytes and neutrophils are increased in the various parts of the lung. Antigen-presenting cells (APCs) usually aid the organization of the recruited lymphocytes into lymphoid follicles, and the presence of oligoclonal lymphocytes recognize, process and present the processed antigen to the naïve lymphocytes (4).

Dendritic cells (DCs), a type of APC, may be involved in the development of COPD, as DCs normally exert a predominant role in the initiation and orchestration of immune responses. DCs are recruited from the circulation and migrate toward epithelial surfaces, where the cells capture antigens and recognize danger signals. Following antigen uptake, DCs migrate to regional draining lymph nodes for antigen presentation. During migration, DCs ingest and process antigens, and upregulate the expression of co-stimulatory molecules at the cell surface, a process known as maturation using the cluster of differentiation (CD)83^+^ cell surface marker (5). In the lymph nodes, DCs present the processed antigen to naive T lymphocytes, resulting in the initiation, suppression or termination of adaptive immune responses if no longer required (6). The airways and lungs contain a rich network of DCs, localized near the epithelial surface. DCs thus normally control immunologic homeostasis, but may function inappropriately in COPD (7).

During the organization of the recruited lymphocytes into lymphoid follicles, chemokines and the corresponding receptors are key determinants of lymphoid tissue organization (8). Among the chemokine receptors (CCRs), CCR7 is an notable receptor with the potential to influence responses in the peripheral tissues and lymph nodes. CCR7 is crucial for the homing of immature T cells and mature DCs to the lymph nodes via dedicated ligands, that is, the lymphoid chemokine (C-C) motif ligand (CCL)19 and CCL21 (9). A previous study observed increased pulmonary CCL19 expression levels in a model of murine COPD, with typical pulmonary lymphoid neogenesis upon CS exposure (10). The determining role of CCR7^+^ in the recirculation of lymphocytes from mucosal tissues is indicated by the finding that CCR7^+^ deficiency results in the marked appearance of ectopic lymphoid follicles in the lung and other mucosal sites (11).

In the present study, the hypothesis that small airways have fewer mature DCs in patients with COPD was analyzed. The study additionally investigated the role of human pulmonary DCs in the pathogenesis of COPD. DC infiltration in the peripheral airways was compared among non-smokers, smokers without airway obstruction and patients with COPD.

## Materials and methods

### Patient tissue sample collection

This study was approved by the medical ethical committee of the Affiliated Hospital of Zunyi Medical College (Zunyi, China). COPD was diagnosed and classified by the Global Initiative for Chronic Obstructive Lung Disease (GOLD) criteria (12). Tissues were obtained from surgical lung resection specimens of the following patients diagnosed with solitary pulmonary lesions: Eight smokers with COPD, eight non-smokers with COPD, eight smokers without COPD and eight non-smokers without COPD (which served as a control group). Lung tissues at the maximum distance from the pulmonary lesion, and without signs of retro-obstructive pneumonia or tumor invasion, were collected by a pathologist. None of the patients who underwent surgery for malignancy were treated with neo-adjuvant chemotherapy. All patients signed informed consent prior to the surgery and were interviewed with regard to smoking habits and medication use. COPD diagnosis and severity were defined using pre-operative spirometry according to the GOLD classification.

### Determination of CD83^+^, CD1a^+^ and CCR7^+^ gene expression levels in COPD and control tissues

The lung tissue samples were ground using liquid nitrogen. Total RNA was extracted from the samples using a human tissue RNA purification kit (Norgen Biotek, Thorold, ON, USA) according to the manufacturer’s instructions. The RNA samples were analyzed for OD260, OD280 and OD230 using an ultraviolet spectrometer (Applied Biosystems, Foster City, CA, USA) to determine RNA purity (OD260/280>1.8 and OD260/230>1.5). Formaldehyde denaturing agarose gel electrophoresis was employed to determine RNA integrity through examination of the 28S to 18S ratio in the RNA samples.

Reverse transcription quantitative polymerase chain reaction (RT-qPCR) analysis was performed to determine the relative expression levels of CCR7^+^ gene transcripts in the COPD and control tissues. For qPCR analysis, the 7900HT Real-Time PCR detection system (Applied Biosystems) was utilized. To generate cDNA for qPCR analysis, SuperScript^®^ III Reverse Transcriptase (Invitrogen, Carlsbad, CA, USA) was employed. To quantify the final cDNA PCR products, SYBR^®^ Green PCR Master Mix (Life Technologies, Grand Island, NY, USA) was used. The conditions for the PCR reactions were as follows: 50°C for 2 min, 95°C for 2 min, followed by 40 cycles of 95°C for 15 sec, 60°C for 30 sec and 72°C for 30 sec. During the 72°C stage, analysis of the SYBR fluorophore for quantification was conducted. The relative expression levels of CCR7 mRNA were calculated by normalization of these levels to GAPDH mRNA levels using the comparative threshold cycle (ct) method, in which fold difference = 2-(Δct of target gene-Δct of reference). The mRNA amplification primers were as follows: CCR7^+^, 5′-GGTGGTGGCTCTCCTTGT CATT-3′ and 5′-GCTTTAAAGTTCCGCACGTCCTT-3′; CD83^+^, 5′-GCTGGAAATGCTGGGCTGA-3′ and 5′-CAT GCAACAGCCTTGTGGTTTAC-3′; CD1a^+^, 5′-CAG GGACATGGGAGCATTG-3′ and 5′-AACAAGTCTGAT GTGGCATTGAA-3′; GAPDH, 5′-CGGTATTTGGTC TATTGGGC-3′ and 5′-TGGAAGATGGTGATGGGATTTC-3′.

### Immunohistochemical detection of mature DCs in COPD tissues

A two-step indirect immunohistochemical method involving unlabeled primary antibodies and labeled secondary antibodies was used to determine the presence of the CD83^+^, CD1a^+^ and CCR7^+^ cell markers in all paraffin-embedded lung tissue samples. The antibodies used were as follows: FITC-conjugated mouse monoclonal antibodies to human to CD3, CD20 and CD14 (#340546; 1:100; BD Biosciences, Franklin Lanes, NJ, USA), FITC mouse monoclonal antibodies to human CD80 (#555683; 1:100; BD Biosciences), FITC mouse monoclonal antibodies to human CD86 (#555657; 1:100; BD Biosciences), FITC mouse monoclonal antibodies to human monoclonal antibodies to HLA-DR (#555811; 1:100; BD Biosciences), FITC-conjugated mouse monoclonal antibody to human CD83 (#MAB1774; 1:200; R&D Systems Inc., Minneapolis, MN, USA), FITC-conjugated mouse monoclonal antibody to human CCR7 (#MAB197-100; 1:50; R&D Systems Inc.) and FITC-conjugated mouse monoclonal antibody to human CD1a (#MAB7076; 1:50; R&D Systems Inc.). Positive-staining cells were assessed using a minimum of five images from each slide. All images were captured using an IPWIN32 catch system (Life Technologies).

### Myeloid DC isolation from lung tissue

BDCA^+^ cells were purified from peripheral blood monocytes (PMCs) using a commercially available isolation kit (Miltenyi Biotec, Bergisch Gladbach, Germany). Briefly, PMCs were first depleted of T cells, monocytes/macrophages and natural killer (NK) cells using anti-CD3, -CD11b and -CD16 beads. Subsequently, this depleted population was incubated with anti-CD4 beads, thus only the CD4^+^ cells were retained. Since all BDCA^+^ cells express CD4, this method allows the pre-enrichment of BDCA^+^ cells and prevents contamination with lymphocytes, monocytes/macrophages and NK cells.

### Flow-cytometric analysis of DCs

For cell surface staining, 200 μl aliquots of bronchial tissue brushings were added to the labeled fluorescence-activated cell sorting tubes. To decrease nonspecific binding, 20 μl normal human immunoglobulin (60 g/l; Intragam^®^; Commonwealth Serum Laboratories, Sydney, Australia) was added to each tube for 20 min at room temperature. After additional incubation for 20 min in the dark, with directly conjugated monoclonal antibodies to surface markers of interest, cells were washed with 0.5% bovine serum albumin in Isoton II (Beckman Coulter, Hialeah, FL, USA; hereafter referred to as wash buffer), centrifuged at 1,500g for 90 sec and the supernatant was discarded. A total of 20 ml wash buffer was added and events were acquired immediately with a FACSCalibur flow cytometer (BD Biosciences) and analyzed with FlowJo software (FlowJo LLC, Ashland, OR, USA). A total of 10,000 events were collected from bronchial brushings.

### Statistical analysis

Statistical analysis was conducted using SPSS 17.0 (SPSS, Inc., Chicago, IL, USA). When evaluating differences in continuous variables among multiple independent groups, the one-way analysis of variance test was used. P<0.05 was considered to indicate a statistically significant difference.

## Results

### Subject characteristics

**Figure 1:**
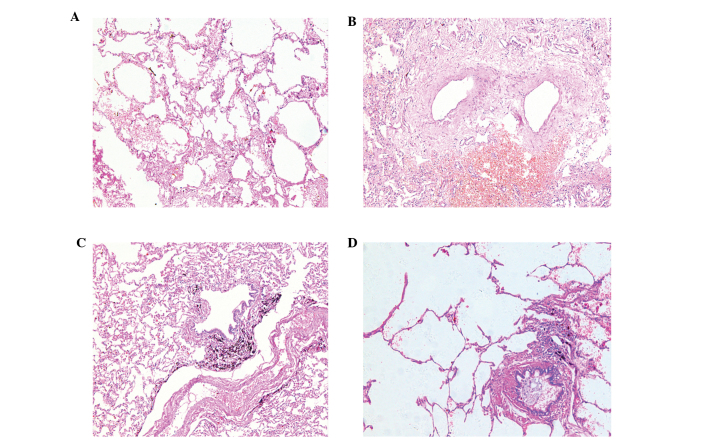
Lung tissue hematoxylin and eosin staining of different groups of patients (magnification, ×200). (A) CS^−^COPD^−^, a transverse section of a small airway of normal appearance with a patent lumen and a relatively thin airway wall with numerous surrounding alveolar attachments. (B) CS^−^COPD^+^, chronic obstructive bronchiolitis with thickening of the airway wall and infiltration with lymphocytes, macrophages and neutrophils. (C) CS^+^COPD^−^, black dust accumulated in the small airway, heavily infiltration with lymphocytes but without narrowing of the airway wall. (D) CS^+^COPD^+^, patients with peribronchiolar destruction of alveolar walls, resulting in the loss of alveolar attachments, airway collapse and enlargement of the air spaces distal to the terminal bronchioles. CS, cigarette smoker; COPD, chronic obstructive pulmonary disease; CS^−^COPD^−^, non-smoker without COPD; CS^−^COPD^+^, non-smoker with COPD; CS^+^COPD^−^, cigarette smoker without COPD; CS^+^COPD^+^, cigarette smoker with COPD.

**Figure 2:**
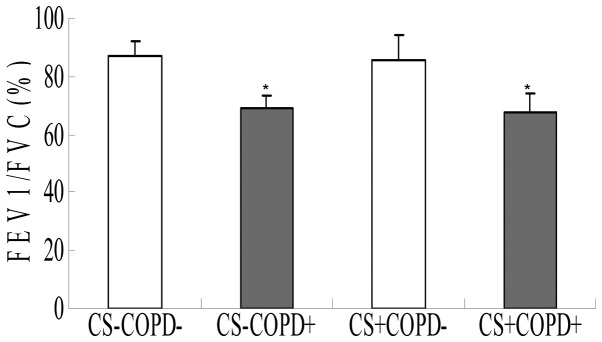
Comparison of lung function (FEV_1_/FVC) of patients in the different groups. Eight individuals were in each experimental group. The results are presented as the mean ± standard deviation. ^*^P<0.05 compared with the CS^−^COPD^−^ group. FEV, forced expiratory volume; FVC, forced vital capacity; CS, cigarette smoker; COPD, chronic obstructive pulmonary disease; CS^−^COPD^−^, non-smoker without COPD; CS^−^COPD^+^, non-smoker with COPD; CS^+^COPD^−^, cigarette smoker without COPD; CS^+^COPD^+^, cigarette smoker with COPD.

Evident chronic bronchitis and emphysema pathological changes were observed in the lung tissues of patients with COPD (Fig. 1). Clinical lung function data from the patients are presented in Fig. 2. As expected from the selection criteria, the forced expiratory volume 1/forced vital capacity ratio was significantly lower in the patients with COPD, as compared with the non-smokers and the asymptomatic smokers (P<0.05). All patients, with the exception of the non-smokers group, were current smokers. No significant differences in age, weight and height among the patients were identified. Furthermore, no substantial differences in smoking history pack-years between the asymptomatic smokers without COPD and those with COPD were detected (P>0.05). The non-smokers group contained approximately equal numbers of males and females but the asymptomatic smokers and the patients with COPD were predominantly males.

### Reduced numbers of mature CD83^+^ and CCR7^+^ DCs, and increased numbers of immature CD1a^+^ DCs in COPD patients

**Figure 3:**
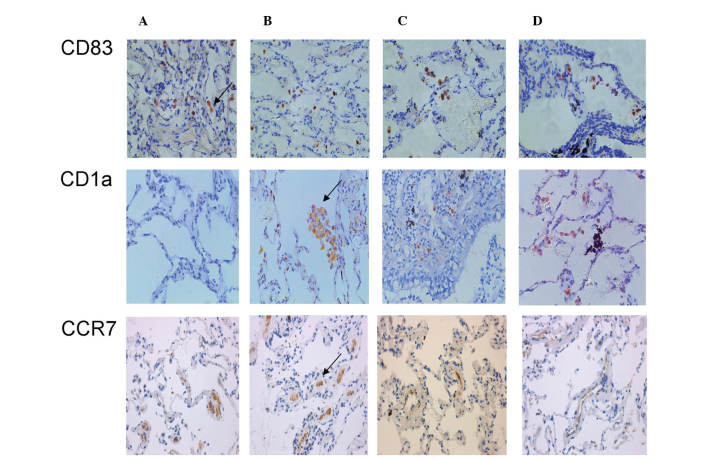
Immunohistochemical analysis of CD83^+^, CD1a^+^ and CCR7^+^ cells in the following groups: (A) CS^−^COPD^−^; (B) CS^−^COPD^+^; (C) CS^+^COPD^−^ and (D) CS^+^COPD^+^. Magnification, ×400. Arrows indicate CD83^+^, CD1A^+^ and CCR7^+^ stained cells. CD, cluster of differentiation; CCR, chemokine receptor; CS, cigarette smoker; COPD, chronic obstructive pulmonary disease; CS^−^COPD^−^, non-smoker without COPD; CS^−^COPD^+^, non-smoker with COPD; CS^+^COPD^−^, cigarette smoker without COPD; CS^+^COPD^+^, cigarette smoker with COPD.

**Figure 4:**
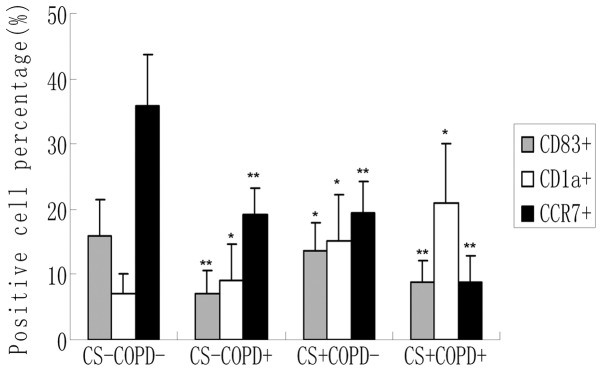
CD83^+^ and CD1a^+^ cells in lung tissue specimens from each group of patients. Data are presented as the mean ± standard deviation, n=8. ^*^P<0.05 and ^**^P<0.01 compared with the control group (CS^−^COPD^−^). CD, cluster of differentiation; CCR, chemokine receptor; CS, cigarette smoker; COPD, chronic obstructive pulmonary disease; CS^−^COPD^−^, non-smoker without COPD; CS^−^COPD^+^, non-smoker with COPD; CS^+^COPD^−^, cigarette smoker without COPD; CS^+^COPD^+^, cigarette smoker with COPD.

Immunohistochemical analysis of CD83^+^, CD1a^+^ and CCR7^+^ DCs revealed that a greater number of CD1a^+^ DCs were detected in virtually all smoker and patients with COPD, whereas CD83^+^ and CCR7^+^ were specific for the control, asymptomatic non-smoker group (Fig. 3). The results of the cell number count (Fig. 4) demonstrated that the numbers of CD83^+^ and CCR7^+^ DCs were significantly reduced, but the numbers of CD1a^+^ DCs were significantly increased in the COPD and smoker groups as compared with the control group (P<0.05).

### Reduced CD83^+^ and CCR7^+^ expression during lung myeloid DC maturation in COPD

**Figure 5:**
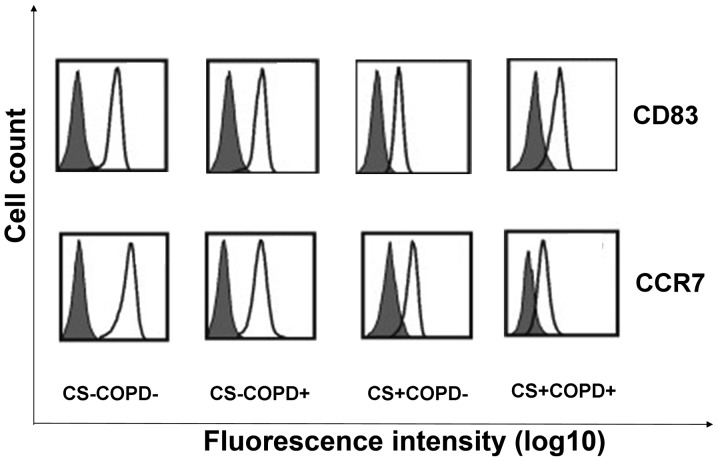
Phenotypic differences during lung myeloid DC maturation. Analysis of cell surface CCR7 and CD83 expression in myeloid DC maturation in the different groups (n=8 each). Representative histograms for each group are shown. Isotype controls are presented as grey-shaded areas and specific antibodies are shown as black lines. CD, cluster of differentiation; CCR, chemokine receptor; CS, cigarette smoker; COPD, chronic obstructive pulmonary disease; CS^−^COPD^−^, non-smoker without COPD; CS^−^COPD^+^, non-smoker with COPD; CS^+^COPD^−^, cigarette smoker without COPD; CS^+^COPD^+^, cigarette smoker with COPD.

The levels of cell surface CCR7 and CD83 expression during myeloid DCs maturation in the four groups was analyzed. Representative histograms for each group are shown in Fig. 5. The CD83 and CCR7 myeloid DC expression levels were reduced as compared with the control group, as detected by flow cytometric analysis.

### Reduced CD83^+^ and CCR7^+^, and increased CD1a^+^ expression levels in lung tissues from COPD patients

**Figure 6:**
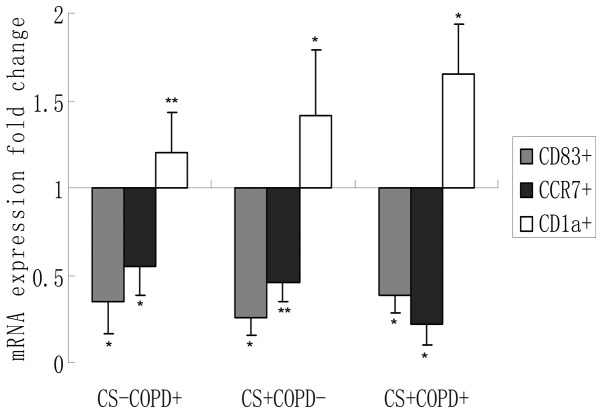
CD83^+^, CD1a^+^ and CCR7^+^ expression levels in lung tissues from the different groups. The results are presented as the mean ± SD, n=8, ^*^P<0.05, ^**^P<0.01, as compared with the control for each marker at a baseline of 1. CD, cluster of differentiation; CCR, chemokine receptor; CS, cigarette smoker; COPD, chronic obstructive pulmonary disease; CS^−^COPD^−^, non-smoker without COPD; CS^−^COPD^+^, non-smoker with COPD; CS^+^COPD^−^, cigarette smoker without COPD; CS^+^COPD^+^, cigarette smoker with COPD.

The expression levels of CD83^+^ and CCR7^+^ mRNA transcripts were significantly lower, and CD1a^+^ expression levels were significantly higher (P<0.05) in COPD lung tissues as compared with lung tissues from non-smokers and smokers without COPD (Fig. 6).

## Discussion

In the present study, the results verified the hypothesis that chronic exposure to CS impairs the normal DC maturation process, and subsequently alters or suppresses normal DC function and interaction with naive lymphocytes, resulting in an imbalance of immunity that may increase the susceptibility of patients with COPD to respiratory infections. A number of markers that have been associated with the maturation of DCs were analyzed. Since active smoking may reduce the numbers of DCs, the data from patients with COPD, from smokers without COPD and from non-smokers were compared.

Traditionally, DCs have been described as key cells in linking the innate and adaptive immune responses, which are both involved in chronic inflammation in COPD (13). The hypothesis that DCs are involved in the development of COPD in smokers has been determined by previous studies. For example, evidence in humans demonstrates that CS induces the recruitment of a large numbers of immature DCs into the small airways of patients with COPD (14). DCs also are ideally localized to initiate an inflammatory reaction in response to inhaled CS. However, few studies concerning the potential of DC involvement in the pathogenesis of COPD have been published. In patients with COPD, significantly increased numbers of small airway langerin-expressing DCs in the bronchoalveolar lavage fluid (BALF) of smokers have been observed (15). An immunohistochemical study demonstrated an increase in the numbers of Langerhans cells in the airways of smokers with COPD, as compared with smokers without COPD and non-smokers (16). Further studies revealed that the numbers of mature DCs were reduced in patients with COPD (17,18). Active smoking in patients with COPD is associated with reduced numbers of bronchial mucosal DCs that express the CD83^+^ maturation marker (19).

The present study quantified airway DC cells in groups of smokers and non-smokers, with and without COPD. The numbers of CD83^+^ DCs were reduced but the numbers of CD1a^+^ DCs were increased in individuals with COPD and those who smoked cigarettes. An early study indicated that the numbers of CD1a^+^ immature DCs are increased in the alveoli and in the BALF of smokers (20). However, normally relatively few CD1a^+^ DCs are detected in human alveoli and these cells comprise <1% BAL cells. Soler *et al* (21) reported no differences in the numbers of CD1a^+^ DCs in the bronchial epithelium between smokers and non-smokers. In concurrence with this finding, another study observed no difference in the numbers of pulmonary langerin-positive immature DCs in small airways between healthy smokers and non-smokers, or between smokers with COPD and ex-smokers (22). By contrast, during analysis of cells in the large airways, a recent study identified mucosal DCs by their ultra-structure in endobronchial biopsies of smokers and ex-smokers with COPD, and demonstrated markedly reduced numbers in those who continued to smoke (16). Furthermore, sputum data have indicated that the numbers of mature CD83^+^ and DC-lysosome-associated membrane glycoprotein 1 (LAMP1) DCs, and the ratios of mature CD83^++^ and mature DC-LAMP1 DCs to total DCs are reduced in current smokers as compared with healthy subjects (23). The reduction in the numbers of mature DCs appears to be associated with smoking status, as a comparable reduction in the number of immunohistologically detected CD83^++^ mature bronchial mucosal DCs has recently been reported in large airways of smokers with asthma, as compared with non-smokers with asthma (24).

In the present study, to further investigate whether the increase in the number of mature DCs in the airways of patients with COPD may be explained by an increase in the numbers of CCR7^+^ cells, CCR7^+^ expression in the human lung at the mRNA level was determined, and the CCR7^+^ expression levels among non-smokers, smokers without COPD and patients with COPD were compared. The data suggest that CS may stimulate these local immune responses by impairing airway DC homing to the lymph nodes, thus promoting local antigen presentation within the airway wall. Pulmonary DC migration to the draining lymph nodes is induced by antigen capture and is characterized by the downregulation of DC antigen capture capacity and the upregulation of DC lymph node homing receptors, predominantly CCR7^+^ (25,26). A consistent and specific association has been detected between reduced CCR7^+^ expression levels in myeloid DCs, and airflow limitation and pulmonary hyperinflation in smokers (27). The possible underlying mechanism that links reduced myeloid DC CCR7^+^ expression levels and airway obstruction may be that impaired homing of myeloid DCs to the lymph nodes results in the accumulation of myeloid DCs in the airways. This accumulation may stimulate local adaptive immune responses, which induce airway remodeling and obstruction. Notably, in the presence of pathogen- and damage-associated molecular patterns, DC migration is accompanied by full DC maturation, a differentiation process characterized by an increase in various cell surface and intracellular molecule expression levels (28,29). Therefore, excessive local adaptive immune responses are key elements in the pathogenesis of COPD. In addition, a previous study reported that CS extracts suppress maturation-associated CCR7^+^ expression in human myeloid DCs *in vitro* (30). Therefore, due to the essential role of CCR7^+^ in the migration of myeloid DCs to draining lymph nodes, CS may reduce the migratory potential of myeloid DCs.

The predominant concern is the definition of which cells detected in the lungs constitute DCs. In mice, pulmonary DCs include CD11c^+^ major histocompatibility complex II (MHC II)^+^ conventional DCs and CD11c^+^ plasmatocytoid DCs (31). Human lung DCs comprise three subsets: Myeloid DC type 1 (BDCA1^+^/MHC II^+^), myeloid DC type 2 (BDCA3^+^/MHC II^+^) and plasmacytoid DC (BDCA2^+^/CD123^+^) (32). The expression of CCR7 or CD83 does not define a cell as a DC. For example, CCR7 may also be expressed by T cells and certain lung cancer cells. In the present study, to validate the results, myeloid DCs were collected from the lung tissue, and CCR7^+^ and CD83^+^ cells were detected through flow cytometry.

Apparently conflicting results are presented in the sparse human literature concerning methodological issues of DC detection, and this may be due to assessment of distinct DC-specific markers, sampling of different anatomic sites, and distinguishing whether the findings are due to smoking status or the disease process itself (33). As an example of the first issue, langerin and CD1a^+^ are considered to be markers of an immature myeloid DC phenotype, whereas CD83^+^ and DC-LAMP are considered markers of mature DCs. In the present study, DC-specific markers in lung tissues samples and the common corresponding CCR7^+^ receptors were selected to improve the study.

As mentioned above, analysis of numerous cell surface markers increased the validity of the present study. However, certain limitations to the present study require further consideration. Although reduced myeloid DC numbers were observed in the patients with COPD, as well as lower CCR7^+^ levels, demonstrating the causal role of DCs in the pathogenesis of COPD is not possible with this approach. For this purpose, *in vivo* animal experiments are required to investigate the effect of overexpression or knockout of DC function on CS-induced inflammation. Such animal models are also required to elucidate the underlying mechanism responsible for the accumulation of DCs in the airways.

In addition, the presence of cancer may have influenced DC infiltration in the small airways, resulting in an enhancement of inflammatory cell recruitment (34). However, primary bronchus carcinoma was the main reason for surgery in all groups, which minimized the risk of confounding factors among groups. Furthermore, recent data have revealed a suppression of DC accumulation in lung cancer, rather than an increase (35). Most importantly, in the present study, the tissue samples removed for analysis were obtained at a distance from the primary pathological lung tissues.

In conclusion, these data indicate that smoking affects the expression profile of function-associated surface molecules on airway myeloid DCs. The involvement of DCs in the pathogenesis of COPD is becoming recognized. The present study provides evidence that reduced CCR7^+^ expression levels on airway myeloid DCs may be associated with airflow limitation in smokers. Future advances in the understanding of pulmonary DCs, combined with recent advances in the pharmaceutical manipulation of DC function, may aid in the identification of novel therapeutic methods with which to prevent or treat COPD more effectively.
